# Editorial: Nutrients and metabolism in fish

**DOI:** 10.3389/fphys.2023.1346380

**Published:** 2023-12-08

**Authors:** Mariana Palma

**Affiliations:** Centre for Functional Ecology, TERRA Associate Laboratory, Department of Life Sciences, University of Coimbra, Coimbra, Portugal

**Keywords:** aquaculture, aquafeeds optimization, bile composition, omics, mitochondrial dynamics, genetic variability, single cell ingredients, environmental impact

Fishes are a very diverse group of aquatic animals with an important role in several and diverse research areas, from ecology to biotechnology and aquaculture. Fish and its derived products also constitute an important source of protein for human nutrition all around the world, being in some regions the major source of animal protein (FAO, 2022). Understanding the fish physiological mechanisms and how they are affected by external factors, such as environmental conditions or diet composition will positively contribute to a broader and comprehensive knowledge of their sophisticated and diverse physiology. The results will certainly provide valuable information and contribute to the development of several research fields.

The relationship between fish nutrition and metabolism is a relevant topic as it affects fish development and health and could then have wider effects on many areas (Tacon et al., 2022), being aquaculture one of the most studied the last few years, specially due to the urgent need to preserve and spare the overexploited wild fisheries stocks (FAO, 2022; Tacon et al., 2022).

Understanding the relationship between nutrient availability, fish metabolism and the consequent effects on fish health, welfare and growth is pivotal to enhance aquaculture production. An efficient metabolism ensures proper utilization of nutrients for homeostasis, energy production and growth. However, metabolism is a dynamic process, influenced by several factors that can ultimately impair fish behaviour and health (Panserat et al., 2019).

In the quest to improve fish production while keeping fish welfare, the sustainable management of aquatic resources and the imperative environmental balance, several studies have been developed, aiming for the preservation of the ocean while meeting the nutritional needs of the growing global population. In this Research Topic, researchers have contributed to a better understanding of how nutrients influence overall fish metabolism, either on diet optimization studies for aquaculture production or when subjected to different environmental conditions.

Bertini et al. and Vasilaki et al. have evaluated the impacts of the incorporation of single-cell protein ingredients on the growth performance of grey mullet (*Mugil cephalus*) and European seabass (*Dicentrarchus labrax*) respectively. The replacement of fishmeal, fish oil and plant-based ingredients by single-cell protein promoted the increase of nutrient digestibility and the gut structure without changing the growth performance parameters. Also regarding diet optimization, Perelló-Amorós et al. evaluated the effects of two exercise levels and two practical diets with different energy levels on gilthead seabream (*Sparus aurata*). It was demonstrated that under voluntary swimming, the fish fed high-protein and high-energy diets showed differences on the expression of the hepatic gene markers associated with energy metabolism and mitochondrial biogenesis. Authors concluded that exercise was able to correct the imbalance promoted by diets with lower protein and higher lipid content.

Kokkali et al. tested experimental diets for Atlantic salmon (*Salmo salar*) smolt with organic and inorganic mineral supplements, incorporated at different levels. Authors observed a trend for a higher growth rate in the organic mineral groups irrespective of the dietary mineral levels. Noteworthy, it was also observed that fish fed the organic supplements presented higher body levels of EPA and DHA and increased slaughter yield, improving their final fillet quality.

Some authors followed the Omics-approaches to answer their research questions, proving the versatility of these methodologies to provide results on diverse areas. The study of Callet et al. resorted to transcriptomics and demonstrated that distinct genotypes of rainbow trout (*Oncorhynchus mykiss*) differently used the plant-based ingredients leading to variable levels of energy production and protein synthesis. The study of Zhao et al. applied the transcriptomics and metabolomics analyses to investigate the molecular mechanisms and its associated regulatory factors linked to vitellogenesis in Sichuan bream (*Sinibrama taeniatus*). Huo et al. developed an integrated transcriptomics and metabolomics approach to assess the lipid metabolism of rice-field eel (*Monopterus albus*), concluding that vitamin A promoted lipid deposition in this species.

The study of Solovyev et al. assessed the elemental composition of fish bile as a marker for ecotoxicological and physiological studies, focusing on the effects of long-term and spatial distribution as well as other biotic and abiotic factors. The authors established that the environmental conditions of a water body, as well as the fish feeding habits, were determinant for the element composition of fish bile. The study contributed to understand which elements were produced by the regular fish metabolism and which resulted from environmental contamination.
